# Investigating the negative relationship between wages and obesity: New evidence from the work, family, and health network

**DOI:** 10.5617/njhe.4720

**Published:** 2018-11-23

**Authors:** MATTHEW J. TROMBLEY, JEREMY W. BRAY, JESSE M. HINDE, ORFEU M. BUXTON, RYAN C. JOHNSON

**Affiliations:** 1 Division of Health and Environment, Abt Associates. 5001 South Miami Blvd., Ste.210, Durham, NC USA; 2 Department of Economics, University of North Carolina at Greensboro, USA; 3 Behavioral Health Financing, Economics, and Evaluation Program, RTI International, USA; 4 Department of Biobehavioral Health, Pennsylvania State University, USA; 5 Division of Sleep Medicine, Harvard Medical School, USA; 6 Department of Social and Behavioral Sciences, Harvard Chan School of Public Health, USA; 7 Sleep Health Institute, Departments of Medicine and Neurology, Brigham and Women’s Hospital, USA; 8 Department of Psychology, Ohio University, USA

**Keywords:** obesity, wages, discrimination, productivity, motherhood, I12, J31, J71

## Abstract

A substantial literature has established that obesity is negatively associated with wages, particularly among females. However, prior research has found limited evidence for the factors hypothesized to underlie the obesity wage penalty. We add to the literature using data from IT workers at a U.S. Fortune 500 firm that provides us with direct measures of employee income and BMI, and health measures that are unavailable in national-level datasets. Our estimates indicate that the wage-obesity penalty among females only occurs among obese mothers, and is not attributable to differences in health or human capital that may be caused by having children.

## 1 Introduction

Obesity is a substantial health issue in Nordic nations. In 2010, rates of obesity ranged from roughly 10–20% across Denmark, Finland, Iceland, Norway, and Sweden (Asgeirsdottir and Gerdtham, 2016).^1^ This was nearly twice the rate observed two decades prior (Asgeirsdottir and Gerdtham, 2016). Numerous studies link obesity to greater rates of absenteeism and presenteeism (i.e. reduced effort while present on the job).^2^ Obesity has also been linked to lower wages in Finland (Brunello and D’Hombres, 2007; Garcia and Quintana-Domeque, 2007), Denmark (Greve, 2008; Garcia and Quintana-Domeque, 2007), Sweden (Dackehag, Gerdtham, and Nordin, 2015) the United States, Great Britain, Canada, China, and Taiwan (see e.g. Register and Williams, 1990; Loh, 1993; Pagan and Davila, 1997; Averett and Korenman, 1996; Averett and Korenman, 1999; Cawley, 2004; Cawley, Grabka, and Lillard, 2005; Harper, 2000; Morris, 2006; Larose et al., 2016; Chu and Ohinmaa, 2016; Garcia and Quintana-Domeque, 2007; Lundborg et al., 2007; Greve, 2008; Shimokawa, 2008; Sabia and Rees, 2012; Lin, 2016), although not all studies find such an effect (Norton and Han, 2008; Brown, 2011; Oreffice and Quintana-Domeque; 2009). Several studies using an instrumental variables approach have ruled out reverse causality – i.e., that lower wages cause obesity.^3^ However the literature has yet to establish the causal mechanism underlying the wage-obesity penalty, nor has it established why the wage-obesity penalty for females is frequently greater than for males.

Our study revisits two previously hypothesized mechanisms – productivity differences between obese and non-obese employees and coworker discrimination against obese employees – using unique data from a single U.S. firm. The data allow us to control for detailed measures of employee health and seniority in the firm and explore whether wages for obese employees vary according to coworker characteristics in a way that may indicate discrimination. We also investigate the previously unconsidered possibility that parenthood may influence the wage-obesity relationship. Given the stylized fact that mothers earn lower wages than females without children, we hypothesize that motherhood may exacerbate the wage-obesity relationship. Such a mechanism could account for gender differences in the wage-obesity penalty.

The following section discusses previous attempts to explain the well-documented negative relationship between wages and obesity. Section 3 introduces the econometric and conceptual models, while Section 4 describes the data. Section 5 reports the results of tests for the three hypothesized confounders. Section 6 provides concluding discussion.

## 2 Literature review

The literature has recently shifted focus from estimating the relationship between obesity and wages to identifying the possible factors(s) that may underlie this relationship (hereafter referred to as the “wage penalty”). One hypothesis is that obese employees are less productive than normal-weight employees for reasons uncaptured by standard wage equations. These differences are typically attributed to differences in human capital accumulation not controlled for by education or experience, or to differences in health.

Support for the human capital mechanism is equivocal. Baum and Ford (2004), find that obese U.S. workers receive smaller wage increases from additional experience, but Atella et al. (2008) find that wage increases from participation in training programs do not differ between obese and normal-weight workers in Europe. The health mechanism has even less empirical support, as studies typically find that controls for health explain little (Johansson et al., 2009; Lundborg et al., 2007) or none (Brunello and D’Hombres, 2007; Baum and Ford, 2004; Atella et al., 2008) of the wage penalty. The primary alternative hypothesis is some type of workplace discrimination against obese workers.^4^ Studies have found that the wage penalty for obese employees is higher in jobs that require more interpersonal skill (Han, Norton, and Stearns, 2009) or socially-oriented jobs (Johar and Katayama, 2012). Conversely, Baum and Ford, 2004 find that obese workers in customer-oriented occupations do not face a higher wage penalty than those outside of such occupations, and no study has differentiated between potential sources of discrimination (e.g., customer, employer, coworker). In each of these studies, obese employees in less-social jobs still faced a significant wage penalty suggesting that discrimination alone cannot fully account for the observed wage penalty.

Our study revisits these hypotheses with clinical measures of health and an indicator of on-the-job human capital accumulation that may better capture productivity than the proxies used in previous studies. Measures of peer BMI also allow us to test for coworker discrimination against obese employees.

We also consider one hypothesis that has not been previously addressed in the literature: that having children may influence the wage-obesity relationship, particularly among females. In many studies the wage penalty is limited to obese females, or obese females face a larger wage penalty than obese males (Harper, 2000; Cawley, 2004; Garcia and Quintana-Domeque, 2007; Greve, 2008; Garcia Villar and Quintana-Domeque, 2009; Johannson, 2009; Han, Norton and Stearns, 2009; Hildebrand and Van Kern, 2010; Johar and Katayama, 2012; Sabia and Rees, 2012; Chu and Ohinmaa, 2016).^5,6^ Moreover, the labor literature has consistently found a wage penalty for motherhood (e.g., Budig and England, 2001; Anderson, Binder, and Krause, 2003; Avellar and Smock, 2003; Gough and Noonan, 2013; Buchmann and McDaniel, 2016). These two penalties may be driven by similar or complementary mechanisms, and in combination, may exacerbate the wage penalty for obese mothers. For example, other research shows that women, on average, spend more time on childcare and other housework than men, frequently leading to lower wages (Hersch and Stratton, 1997; Carlson and Lynch, 2017). Because the physical and psychological stress associated with motherhood may be positively associated with obesity and negatively correlated with work productivity, the wage penalty may be amplified for working mothers.

Moreover, in experimental job applications, mothers were perceived to be less dependable or committed to work than non-mothers, and judged to be less competent than fathers (Fuegen et al., 2004; Heilman and Okimoto, 2008). Multiple studies have also found working mothers to have less “interpersonal appeal,” indicating that fewer respondents would want them as friends or coworkers (Okimoto and Heilman, 2012). Perceptions of mothers as less dedicated, skilled, or likeable may be exacerbated among obese mothers, since obese employees are perceived as lazy, less disciplined, and less competent than non-obese employees (Rudolph et al., 2009; Bellizzi and Norvell, 1991; Klassen, Jasper, and Harris, 1993; Klesges et al., 1990; Larkin & Pines, 1979; Rothblum, Miller, and Garbutt, 1988; Larwood, 1995). We explore the motherhood, productivity, and discrimination hypotheses in Section 5.

## 3 Conceptual and econometric model

We model wages as

$${\text{W}}_{\text{ij}}=\text{exp}({\text{X}}_{\text{ij}}^{\prime }\;\beta +\lambda_{1}{\text{OV}}_{\text{i}}+\lambda_{2}{\text{OB}}_{\text{i}}+\xi_{\text{ij}})$$

where X is a vector of standard wage covariates, OV is a binary indicator for overweight, OB is a binary indicator for obese, and ξ_ij_ is an error term that may vary at the individual or workgroup level.^7^ This equation estimates the relationship between wages and obesity. We assume that initial estimates of λ_2_ will be biased by omitted variables (e.g., health or human capital) that are jointly correlated with obesity and wages. To test the productivity hypothesis, we add new variables that proxy for productivity through either the health or human capital mechanisms. If these variables are jointly correlated with wages and obesity, then including them in the model should reduce omitted variables bias, shifting the estimated obesity coefficient (λ_2_) closer to the true relationship between wages and obesity. Although prior studies have adopted this approach, ours is the first to use a generalized Hausman test to determine whether the shift in the obesity coefficient is statistically significant, which will indicate whether a given set of variables is a significant confounder of the wage-obesity relationship.

To test the discrimination and parenthood hypotheses, we use interaction terms to allow the wage-obesity relationship to vary by regime. Research has shown that individuals are more likely to discriminate publicly when it is considered socially acceptable to do so (Crandall, Eshelman, and O’Brien, 2002) and that individuals are more likely to adopt negative stereotypes when those stereotypes are expressed or supported by their peer group (Haslam et al., 1996, 1999; Sechrist and Stangor, 2001; Stangor et al., 2001; Wittenbrink & Henly, 1996) Evidence also suggests that perceptions of racial and/or gender discrimination become more likely the smaller the proportion of a group is comprised of an employee’s gender or racial group (Hirsh and Kornrich, 2008; Stainback, Ratliff, and Roscigno, 2011; Stainback and Irvin, 2012). Thus, as the proportion of obese individuals decreases, it may become more acceptable to discriminate, or more likely for negative stereotypes against obese workers to spread. We hypothesize that if there is the potential for discrimination against obese individuals within an organization, we should see discrimination increasing as the proportion of obese employees in a workgroup decreases, leading to lower wages for obese employees with fewer obese coworkers.

To test the discrimination mechanism, we calculate the proportion of each workgroup that is obese and include an indicator for each quartile of this proportion as well as overweight and obese-indicator interactions with each quartile.^8^ The fourth quartile (>75% of the workgroup is obese) was the omitted category, similar to the approach used by Stainback, Ratliff, and Roscigno (2011) and Stainback and Irvin (2012) to measure perceptions of gender and racial discrimination.

To test the parenthood hypothesis, we include an interaction between total number of children and obesity, to allow the wage-obesity relationship to vary between parents and non-parents. This allows us to interpret the wages of obese parents relative to normal-weight parents, and the wages of obese parents relative to obese non-parents.

## 4 Data

The data were obtained from the Work, Family, and Health Study (King, Karuntzos, Casper, et al., 2012; Bray, Kelly, Hammer, et al., 2013). Two American firms in separate industries participated in the study and we focused on the telecommunications firm, which provided administrative salary data. We use a cross-section of data from this firm, collected prior to an intervention unrelated to the current study.

The WFHS sample examined here is limited to full-time, permanent employees, each of whom belongs to one of 106 “work groups.”^9^ Work groups are collections of employees who report to the same manager and may frequently collaborate. Employees operate at one of thirteen sites which are located in one of two urban locations in two separate states. The mean work group size in the sample is approximately 12, and the average work site hosts about 58 employees.

Employees were classified by the firm’s human resources department as support personnel (e.g. network administrators, administrative assistants) or core personnel (those directly involved in the firm’s core business). Work groups were classified as one of four occupational functions assigned by the study team. The support/core and occupational categories are utilized as additional controls in the wage equation.

Consistent with the literature, the dependent variable of interest is a continuous measure of hourly wages, (W_ij_) the ratio of annual income to annual hours. Annual income is obtained from administrative records. Annual hours are obtained by multiplying self-reported average weekly hours by 52 and subtracting the number of vacation or sick leave hours used that year. BMI was constructed from measures of height and weight collected by trained data collectors. Direct measure of salary and BMI reduces measurement error in the dependent variable and variable of interest, limiting the bias in our estimates compared to past studies.^10^

Five male observations and six female observations (1.0% and 1.9% of the original sample, respectively) were dropped due to missing BMI. Thirty-seven male observations (7.6%) and eighteen female observations (5.8%) were dropped due to missing salary data. Among both genders, missing BMI is uncorrelated with other strong predictors of BMI such as age, quadratic age, or education, suggesting that BMI is missing at random (MAR) (Rubin, 1976). Missing BMI is also uncorrelated with wages or any of the other covariates in the primary regression specification, suggesting these data are missing completely at random (MCAR). No observation had missing BMI and missing salary data.

Missing salary data are assumed to be MAR. Employees provided self-reported annual income in increments of $10,000. Categorical income was uncorrelated with missing salary data among females, as was BMI. Among males, 11 of 12 categories were uncorrelated with missing salary, but annual income over $150,000 was correlated with missing salary data. However, this only affected 3 missing observations. Though the MAR assumption partially fails for males, the general lack of results among males should be robust to any information that might have arisen from those three observations. We revisit the implications of the missing data in the limitations discussion.

Due to the small sample, extra attention is paid to outliers that may have an undue influence on the parameter estimates, and we drop any observation that fulfills two conditions: belonging to the top or bottom 1-percentile of BMI, and belonging to the top or bottom 1-percentile of log hourly wages. This results in two males and one female being dropped, leaving a final sample of 448 men and 287 women. Summary statistics are provided in Tables 1a (females) and 1b (males).

Baseline controls for the hourly wage include age, squared age, tenure with the firm, tenure squared, education, race, nativity, married/cohabitating status, number of children, occupation (at the workgroup level), an indicator to differentiate between support and core employees, and indicators for state and worksite.^11^

The data include additional proxies for health and human capital that allow us to account for differences in productivity that may remain after accounting for the standard controls used in the literature. Core personnel in the data are differentiated between “staff” and “senior” level workers, where senior status indicates one or more promotions from the staff levels. This measure may better capture human capital accumulation. We proxy for health using several measures. The first is a measure of physical function, rated on a scale from 0–100. A score of 100 reflects full functionality (able to run and play sports) and 0 is barely functional (health severely limits everyday activities). Obesity is expected to reduce physical function, potentially diminishing productivity.

Another way in which obesity may result in reduced productivity is if obesity disrupts sleep. We include a control for self-reported “loud snoring,” which can predict sleep apnea (Vgontzas et al., 1994; Maislin et al., 1995). Sleep apnea is strongly correlated with obesity, which in turn has been associated with decreased cognitive function (Engleman and Douglas, 2004; Ulfberg et al., 1996; Vgontzas et al., 1994). Obesity is also significantly correlated with insomnia, which may impair concentration and memory (Watson, et al., 2006; Janson, et al., 2001). Consistent with Buxton et al. (2012) we create an indicator for employees reporting waking in the middle of the night three or more times per week, signifying that insomnia symptoms have resulted in sleep deficiency.

Lastly, we include four proxies for cardiovascular health including blood serum levels of C-reactive protein (CRP), a biomarker for inflammation; cholesterol ratio; hypertension; and heart rate.^12^ All four of these measures capture elements of risk for cardiovascular disease, as well as overall cardiovascular fitness, and were obtained by trained data collectors rather than self-reported, with higher levels indicating diminished health. Higher measures of cholesterol and blood pressure/hypertension have been linked to additional employee absenteeism and/or presenteeism. Increased cardiovascular fitness has been linked to less employee absenteeism and/or presenteeism.^13,14^

Both males and females have an average BMI of 28, about 1.5 units higher than the national average. Approximately 31 percent of females and 43 percent of males are overweight, while about 30 percent of males and 31 percent of females are obese.^15^ Obese employees of both sexes are less healthy across all measures, with the exception of insomnia symptoms among females. Obese employees of both sexes are also more likely to have achieved senior status, although this may be because they are older and have longer tenure on average.

## 5 Results

Results from the initial model containing only standard control variables are reported in Table 2. Among females, overweight employees earn approximately 5.7 percent less than those in the normal-weight category (p<0.10), while obese employees earn roughly 7.9 percent less (p<0.05), on average. Neither overweight nor obese males earn significantly different wages compared to their normal-weight coworkers. The lack of a significant wage-obesity relationship for males is consistent with the literature, which has found mixed results regarding the wage-obesity relationship among men. The controls are statistically insignificant, or significant in the expected direction, with one exception. Among females, tenure is negatively correlated with wages, which may indicate that newer hires are paid more than individuals with long tenures with the firm.

For the sake of brevity, only results for obese workers relative to normal-weight workers will be reported in results tables moving forward. All models include indicators for overweight, and interaction models include an overweight interaction term in addition to the reported obese interaction term. Normal-weight employees are the reference group in all models.

Table 3 shows the effect of adding an indicator distinguishing between staff and senior employees, adding health proxies, and including both sets of variables in the model. Unreported coefficients indicate that senior status is associated with 17% higher wages for males and 14% among females, relative to staff, suggesting that it is a strong proxy for productivity. The health variables are jointly significant among males (p<0.05), but not females. This may suggest that among females, health is a weaker proxy for unobserved productivity.

Among males, controlling for senior status significantly reduces the small wage premium by 2.1 percentage points from the baseline model (p<0.10), while controls for health dramatically increase the wage premium by 4.8 percentage points (p<0.01), suggesting that although obese males do not face a wage penalty, their wages could be significantly higher if their health improved. The wage penalty among females remains significant in both models that include health controls. Likewise, the shift in the obesity coefficient is not statistically significant in either case, and accounts for only about 1/5 of the wage penalty. This suggests that the wage penalty among females is not substantially driven by differences in seniority.

Results of the discrimination model are reported in Table 4 for females and Table 5 for males. Table 4 shows that there is no evidence of discrimination against obese females. None of the obesity-quartile interactions are statistically significant and two are of the opposite sign we would expect if discrimination was occurring. There is also no rank order in the results. Obese females in workgroups with less than 25% obese employees earn the same as obese females in workgroups with 50–75% obese employees, and earn more than obese females in workgroups with 75%−100% obese employees.

We also find no evidence of discrimination against obese males. The net effect of obesity in the lowest three quartiles follows the opposite rank order we would expect to see if discrimination was present. Shifting from a workgroup with the most to fewest obese employees has no effect on wages, while shifting from the fourth to third quartile –where obese employees still comprise the majority– significantly decreases wages.

Ultimately, we cannot rule out the possibility of discrimination as we cannot control for every possible mechanism of transmission (e.g., supervisors giving lower ratings or fewer advancement opportunities to obese employees). However, we find no evidence that the wages of obese employees vary depending on the proportion of obese workers in the workgroup suggesting that discrimination is not occurring through this channel.

The final set of analyses investigates the possibility that the wage-obesity dynamic is correlated with parenthood. To test this we augment the combined health and human capital model with interactions between the number of children and the overweight/obesity indicators. The net effect of being obese and a parent is calculated using the average number of children in the sample (i.e. the effect of being obese with an average number of children). We use the median/mode of two children since nobody in the sample has the mean number of children. Results are presented in Table 6.

The base obesity coefficient in this model is 3.5 percent. Although not significant, this estimate suggests that obese females without children earn a wage premium relative to normal-weight females. Among normal-weight mothers, having children is uncorrelated with wages. However, relative to obese females with no children, obese mothers earn 8.3 percent lower wages (p<0.01). Relative to normal-weight mothers, obese mothers earn 6.7 percent lower wages (p<0.05). These results indicate that obese females without children and normal-weight mothers do not face a wage penalty, while obese mothers face a substantial wage penalty.

To see if single parenthood may be driving the wage penalty for obese mothers we estimated a new specification with a triple interaction between obesity, marriage/cohabitation, and children. Results (Table 7) are limited to females since there is no wage-effect of children among males.

Obese single mothers face an average wage penalty of 7.6 percent per child (p<0.05), while obese mothers who are married or cohabitating are estimated to face a wage penalty of 4.9 percent per child (p<0.10). These two wage penalties are not significantly different from one another as demonstrated by the obesity-marriage-children interaction coefficient in Table 7. Although obese single mothers may bear a higher wage penalty per child, obese mothers with a spouse or partner at home still face a significant penalty of nearly 5 percent, indicating that single parenthood may exacerbate but does not solely drive the obesity penalty among mothers in our sample.

The results also do not appear to be driven by higher BMI among obese mothers relative to obese females without children. Obese mothers average 2.1 fewer BMI units than obese non-mothers (p<0.10). The results are also not driven by major differences in the underlying distribution of children between normal-weight and obese mothers. On average, obese mothers have only 1/3 more children than normal-weight mothers and the maximum number of children in both groups is five.

Although we find robust evidence of a wage penalty associated with motherhood, we caution that our model cannot explicitly identify motherhood as causing the wage-obesity relationship. Motherhood may serve as a proxy for some unobserved factor that ultimately mediates the wage-obesity relationship.

## 6 Limitations and discussion

Consistent with previous literature, we find a negative relationship between wages and obesity among females but not males. Proxies for health and human capital only account for about 1/5 of the wage-penalty and this reduction is statistically insignificant. We find no evidence of coworker discrimination against obese employees but find that obese mothers incur a 5.1 percent wage penalty per additional child (p<0.01), while obese women without children incur no penalty. Obese mothers with two children earn 6.7 percent less than normal-weight mothers with two children (p<0.05), and 8.3 percent less than obese females without children (p<0.01). Although more severe among single mothers, the wage penalty occurs for obese mothers regardless of marital status, indicating that single-parenthood cannot be the primary driver. The results are also not driven by irregularities in the distribution of children among obese females, nor by higher average BMIs of obese mothers relative to obese females without children.

Our results come with several limitations. The small sample size reduces the precision of our estimates. However, none of our key conclusions are limited by statistical precision. Results are significant despite the small sample, or the direction and magnitude of the effects are sufficient for us to rule out a hypothesized confounder of the wage-obesity relationship.

Although roughly 9% of the original sample is missing for both males and females, we argue that the data are MAR, and that the essential relationships between wages, obesity, and the hypothesized confounders can be retrieved conditional on the covariates in our model. As a robustness check, we imputed salary data from self-reported categorical income and re-ran all models with the imputed wage data.^16^ As a second robustness check, we re-ran all models, weighted for the inverse probability of missing salary or BMI. All of our estimates and conclusions are robust to these two approaches.

Our estimates are cross-sectional which means we cannot make any causal attributions. However, we did not intend to produce causal estimates of the wage-obesity relationship, but rather to identify potential factors confounding the wage-obesity relationship. Although our results would be strengthened by longitudinal data, the failure of the productivity and discrimination mechanisms to account for the wage-obesity penalty is consistent with the prior literature. The finding that the wage-obesity penalty is limited to mothers provides a novel correlation to follow-up on with longitudinal datasets.

Despite these limitations our data come with several advantages that bolster the internal validity of our findings. First, direct measures of salary and BMI help to reduce bias from (potentially systematic) measurement error. Second, our measures of health and human capital may be better (or at least broader) proxies for employee productivity than those used previously in the literature. Third, recent evidence has suggested that wage inequality is driven primarily by between-firm differences in spending rather than within-firm (Song et al., 2016). By limiting the sample to a single firm, we are able to eliminate potential sources of confounding that are not accounted for by industry-level controls in national datasets such as hiring discrimination against obese employees by high-wage firms, or selection of obese employees into low-wage firms. This bolsters our finding that within-firm differences in productivity or coworker discrimination against obese employees are not driving the wage-obesity relationship for this particular sample. However, this may limit the generalizability of these findings.

We conclude that motherhood is a potential explanation as to why females are estimated to face a wage-obesity penalty more consistently than males. However, our model can only note this as a promising correlation for follow-up and cannot identify motherhood as the causal mechanism of the wage-obesity relationship. It remains noteworthy that we do not find an obesity penalty for non-mothers, nor a motherhood penalty for non-obese females. This suggests that in our sample, neither motherhood nor obesity is a sufficient condition for females to face lower wages as observed in previous literature. The results do not appear to be driven by the potential effects of children on human capital accumulation or health since these factors are conditioned for in the motherhood models. Future research should seek to replicate these results using national-level and/or longitudinal datasets. If this pattern persists in other samples, understanding why the obesity penalty is specific to mothers may help policymakers identify the best ways to accommodate working mothers, in ways that could not only improve their wages, but potentially their health as well.

## Figures and Tables

**Table 1a:** Summary statistics (Female)

		Full Sample (n = 287)		Obese (n = 89)		Normal Weight (n=108)	
		Mean	Standard Deviation	Mean	Standard Deviation	Mean	Standard Deviation
**Standard Variables**	BMI	28.28	6.66	36.15***	5.74	22.43	1.76
	Overweight	31.36%	46.48%				
	Obese	31.01%	46.33%				
	Hourly Wage	38.76	7.86	36.75***	7.97	40.47	7.53
	Log Hourly Wage	3.64	0.21	3.58***	0.21	3.68	0.19
	Married or Cohabitating	71.08%	45.42%	52.81%***	50.20%	75.00%	43.50%
	Total Number of Children	1.63	1.26	1.72	1.37	1.49	1.20
	Nonwhite	24.74%	43.22%	17.98%*	38.62%	27.78%	45.00%
	Born Abroad	19.51%	39.70%	7.87%***	27.07%	27.78%	45.00%
	College Graduate (4-year)	66.55%	47.26%	53.93%***	50.13%	75.93%	42.95%
	Age	46.90	8.38	48.29***	7.70	45.24	9.13
	Tenure with Firm (years)	16.11	9.98	17.92**	10.23	15.02	9.94
	Support Personnel	5.57%	22.98%	8.99%	28.76%	4.63%	21.11%
**Productivity Controls (Z)**	Staff	36.59%	48.25%	39.33%	49.12%	33.33%	47.36%
	Senior	57.84%	49.47%	51.69%*	50.25%	62.04%	48.76%
	Physical Function	92.52	13.52	86.83***	17.89	96.04	7.60
	Loud Snoring	25.44%	43.63%	40.45%***	49.36%	11.11%	31.57%
	Insomnia	54.70%	49.87%	57.30%	49.74%	50.00%	50.23%
	C-Reactive Protein^a^	3.12	4.33	5.83***	6.28	1.64	2.13
	Cholesterol Ratio^b^	3.87	1.09	4.23***	1.17	3.61	0.91
	Hypertension	26.13%	44.01%	39.33%***	49.12%	13.89%	34.74%
	Resting Heart Rate	72.55	11.03	73.95*	12.15	71.48	10.61

Notes:

a: C-Reactive Protein concentration is measured in mg/L. For reference, CRP levels below 1.0 are considered low-risk for heart disease, 1.0–2.99 is considered average risk, and greater than 3.0 is high risk.

b: A cholesterol ratio below 3.5 is considered optimal, 3.5–5 is normal, and >5 is considered high.

* Indicates obese employees are significantly different from normal-weight employees at the 0.1 level.

** p < 0.05,

*** p < 0.01.

**Table 1b:** Summary statistics (Male)

		Full Sample (n = 448)		Obese (n = 134)		Normal Weight (n=122)	
		Mean	Standard Deviation	Mean	Standard Deviation	Mean	Standard Deviation
**Standard Variables**	BMI	28.10	4.92	33.76*	4.39	23.04	1.56
	Overweight	42.86%	49.54%				
	Obese	29.91%	45.84%				
	Hourly Wage	41.02	8.85	42.13**	9.24	40.30	8.52
	Log Hourly Wage	3.69	0.23	3.72*	0.23	3.67	0.22
	Married or Cohabitating	84.82%	35.92%	85.82%	35.01%	81.97%	38.60%
	Total Number of Children	1.64	1.43	1.83***	1.50	1.33	1.25
	Nonwhite	29.24%	45.54%	17.16%***	37.85%	45.90%	50.04%
	Born Abroad	31.70%	46.58%	16.42%***	37.18%	48.36%	50.18%
	College Graduate (4-year)	83.48%	37.18%	73.13%***	44.49%	91.80%	27.54%
	Age	45.13	9.37	46.98***	9.07	43.25	9.42
	Tenure with Firm (years)	12.13	8.17	12.66**	8.12	10.89	7.33
	Support Personnel	6.92%	25.41%	4.48%**	20.76%	10.66%	30.98%
**Productivity Controls (Z)**	Staff	33.26%	47.17%	34.33%	47.66%	32.79%	47.14%
	Senior	59.82%	49.08%	61.19%	48.91%	56.56%	49.77%
	Physical Function	95.24	10.24	92.91***	12.33	96.99	6.59
	Loud Snoring	32.81%	47.01%	39.55%***	49.08%	23.77%	42.74%
	Insomnia	45.09%	49.81%	51.49%	50.17%	37.70%	48.66%
	C-Reactive Protein^a^	1.80	2.27	2.42***	2.84	1.00	1.06
	Cholesterol Ratio^b^	4.46	1.32	4.58**	1.08	4.29	1.67
	Hypertension	36.83%	48.29%	52.24%***	50.14%	24.59%	43.24%
	Resting Heart Rate	69.69	11.36	73.41***	12.00	68.22	10.79

Notes:

a: C-Reactive Protein concentration is measured in mg/L. For reference, CRP levels below 1.0 are considered low-risk for heart disease, 1.0–2.99 is considered average risk, and greater than 3.0 is high risk.

b: A cholesterol ratio below 3.5 is considered optimal, 3.5–5 is normal, and >5 is considered high.

* Indicates obese employees are significantly different from normal-weight employees at the 0.1 level.

** p < 0.05,

*** p < 0.01.

**Table 2:** The relationship between wages and obesity with standard control variables (Model 1)

	Female (n = 287)	Male (n = 448)
Obese	−0.079** (0.032)	0.037 (0.025)
Overweight	−0.057* (0.032)	−0.004 (0.022)
Married or Cohabitating	0.024 (0.030)	0.015 (0.026)
Total Number of Children	−0.005 (0.011)	0.007 (0.008)
Nonwhite	−0.042 (0.029)	−0.027 (0.026)
Born Abroad	0.025 (0.033)	0.041 (0.027)
College Graduate (4-year)	0.062** (0.024)	0.057* (0.033)
Age (10 years)	0.323*** (0.118)	0.345*** (0.090)
Age Squared (100 squared years)	−0.031** (0.012)	−0.033*** (0.010)
Tenure with Firm (10 years)	−0.148** (0.062)	−0.022 (0.044)
Tenure Squared (100 squared years)	0.041*** (0.015)	0.012 (0.011)
Core (Staff and Senior)	0.152** (0.064)	0.227*** (0.035)

Note: Models estimated using generalized linear model with log link. Dependent variable is hourly wage. The overweight and obese coefficients report wages relative to normal-weight employees (BMI < 25). The model includes indicators for worksite, state, and job category. Standard errors are clustered at the work group level and reported in parentheses.

* Significant at the 0.1 level,

** Significant at the 0.05 level,

*** Significant at the 0.01 level.

**Table 3:** Productivity differences as a potential factor underlying the wage – obesity relationship

		Female	Male
Baseline	Obese	−0.079** (0.032)	0.037(0.025)
	Δ		
Human Capital	Obese	−0.064** (0.032)	0.016 (0.023)
	Δ	0.015^#^ [0.082]	−0.021^#^ [0.053]
Health	Obese	−0.065* (0.036)	0.085*** (0.028)
	Δ	0.013 [0.440]	0.048^#^ [0.000]
Combined	Obese	−0.052 (0.036)	0.052** (0.026)
	Δ	0.027 [0.163]	0.015 [0.311]

Note: Models estimated using generalized linear model with log link. Dependent variable is hourly wage. Δ indicates the change in the obesity coefficient caused by including productivity proxies. Models control for employee’s state, site, age, age2, race, nativity, marital status, number of children, tenure with the firm (in years), tenure2, and job category, unless reported otherwise. The human capital model includes indicators for “staff” and “senior” employees. The health model includes controls for physical function, loud snoring, insomnia symptoms, log c-reactive protein concentration, log cholesterol ratio, resting heart rate, and hypertension. All models also contain an indicator for overweight. The normal-weight category therefore serves as the point of reference for all obesity and obesity-interaction coefficients. Standard errors clustered at the work group level and reported in parentheses. p-value for chi-squared statistic from generalized Hausman test reported in brackets.

# Signifies that the obesity coefficient is significantly different from the Baseline coefficient estimate.

* Significant at the 0.1 level,

** Significant at the 0.05 level,

*** Significant at the 0.01 level.

**Table 4:** Estimated wage effects of workgroup composition among females

	Combined	Obese Distribution
**Coefficients**		
Obese	−0.052 (0.036)	−0.099 (0.069)
Quartile 1 (0–25% obese)		−0.008 (0.050)
Quartile 2 (25–50% obese)		0.054 (0.049)
Quartile 3 (50–75% obese)		−0.013 (0.060)
Obese X Quartile 1		0.097 (0.078)
Obese X Quartile 2		−0.014 (0.078)
Obese X Quartile 3		0.094 (0.074)
**Net Effects**		
Obese and Quartile 1		0.089 (0.054)
Obese and Quartile 2		0.041 (0.053)
Obese and Quartile 3		0.081 (0.055)

Note: Models estimated using generalized linear model with log link. Dependent variable is hourly wage. Models control for employees’ state, site, age, age2, race, nativity, marital status, number of children, tenure with the firm, tenure2, job category, indicators for staff and senior, an indicator for loud snoring, an indicator for insomnia symptoms, as well as log cholesterol ratio, log CRP plasma concentration, hypertension, and heart rate. All models also contain an indicator for overweight, and overweight is interacted with the quartile flags in both the obesity and gender models. The normal-weight category therefore serves as the point of reference for all obesity and obesity-interaction coefficients.

Net effects refer to the estimated counterfactual change in wages for an obese employee in the fourth quartile (75%−100% obese or 75%−100% same-gender) moving into the first, second, or third quartile of the obesity or gender distribution.

Standard errors clustered at work group level and reported in parentheses.

* Significant at the 0.1 level,

** Significant at the 0.05 level,

*** Significant at the 0.01 level.

**Table 5:** Estimated wage effects of workgroup composition among males

	Combined	Obese Distribution
**Coefficients**		
Obese	0.052** (0.024)	0.100** (0.046)
Quartile 1 (0–25% obese)		0.002 (0.036)
Quartile 2 (25–50% obese)		0.032 (0.038)
Quartile 3 (50–75% obese)		0.164*** (0.061)
Obese X Quartile 1		−0.006 (0.053)
Obese X Quartile 2		−0.047 (0.053)
Obese X Quartile 3		−0.290*** (0.072)
**Net Effects**		
Obese and Quartile 1		−0.004 (0.037)
Obese and Quartile 2		−0.016 (0.035)
Obese and Quartile 3		−0.126*** (0.039)

Note: Models estimated using generalized linear model with log link. Dependent variable is hourly wage. Models control for employees’ state, site, age, age^2^, race, nativity, marital status, number of children, tenure with the firm, tenure^2^, job category, indicators for staff and senior, an indicator for loud snoring, an indicator for insomnia symptoms, as well as log cholesterol ratio, log CRP plasma concentration, hypertension, and heart rate. All models also contain an indicator for overweight, and overweight is interacted with the quartile flags in both the obesity and gender models. The normal-weight category therefore serves as the point of reference for all obesity and obesity-interaction coefficients.

Net effects refer to the estimated counterfactual change in wages for an obese employee in the fourth quartile (75%−100% obese or 75%−100% same-gender) moving into the first, second, or third quartile of the obesity or gender distribution.

Standard errors clustered at work group level and reported in parentheses.

* Significant at the 0.1 level,

** Significant at the 0.05 level,

*** Significant at the 0.01 level.

**Table 6:** Estimated wage effects of children

		Combined	Children
**Females**	**Coefficients**		
	Obese	−0.052 (0.036)	0.035 (0.044)
	Children		0.009 (0.013)
	Obese X Children		−0.051*** (0.016)
	**Net Effects**		
	Obese and parent		−0.048 (0.046)
	Δ parent if obese		−0.083*** (0.029)
	Δ obese if parent		−0.067** (0.033)
**Males**	**Coefficients**		
	Obese	0.052** (0.026)	0.052 (0.033)
	Children		0.017 (0.013)
	Obese X Children		−0.001 (0.012)
	**Net Effects**		
	Obese and parent		0.082*** (0.029)
	Δ parent if obese		0.031* (0.016)
	Δ obese if parent		0.049* (0.027)

Note: Models estimated using generalized linear model with log link. Dependent variable is hourly wage. Models control for employees’ state, site, age, age2, race, nativity, marital status, number of children, tenure with the firm, tenure2, job category, indicators for staff and senior, an indicator for loud snoring, an indicator for insomnia symptoms, as well as log cholesterol ratio, log CRP plasma concentration, hypertension, and heart rate. All models also contain an indicator for overweight, and overweight is interacted with children. The normal-weight category therefore serves as the point of reference for all obesity and obesity-interaction coefficients.

The Children column presents results for a model interacting children with obesity.

Obese X Children refers to the interaction between obesity and children

Δ parent if obese is the net effect of an obese employee shifting from a non-parent to one with two children. Δ obese if parent is the net effect of a parent with two children shifting from normal-weight to obese.

* Significant at the 0.1 level,

** Significant at the 0.05 level,

*** Significant at the 0.01 level.

**Table 7:** Estimated wage effects of children for single and married parents – female

**Coefficients**	
Obese	0.018 (0.055)
Children	0.024 (0.027)
Obese X Children	−0.076** (0.032)
Obese X Married X Children	0.027 (0.043)
**Net Effects**	
Δ children if obese and single	−0.104*** (0.036)
Δ children if obese and married	−0.079* (0.046)
Δ married if obese and parent	0.068 (0.045)
Δ married if obese and no kids	0.043 (0.064)

All models estimated using a generalized linear model with log link. Dependent variable is hourly wage. Models control for employees’ state, site, age, age2, race, nativity, marital status, number of children, tenure with the firm, tenure2, job category, indicators for staff and senior, an indicator for loud snoring, an indicator for insomnia symptoms, as well as log cholesterol ratio, log CRP plasma concentration, hypertension, and heart rate. All models also contain an indicator for overweight and overweight interaction terms. The normal-weight category therefore serves as the point of reference for all obesity and obesity-interaction coefficients. Children gives the change in wages of having a child for a normal-weight, single parent.

Obese X Children refers to the interaction between obesity and children

Obese X Children X Married refers to the interaction between obesity, married/cohabitating, and children.

Δ children if obese and single presents the net effect of a single, obese female with no children switching to a regime where she has two children

Δ children if obese and married presents the net effect of a married, obese female with no children switching to a regime where she has two children.

Δ married if obese and parent presents the net effect of a single obese female with two children switching to a married regime.

Δ married if obese and no kids presents the net effect of a single obese female with no children switching to a married regime.

* Significant at the 0.1 level,

** Significant at the 0.05 level,

*** Significant at the 0.01 level.

## Footnotes

1 Body Mass Index (BMI) is the standard scale for establishing a healthy weight to height ratio. BMI equals the ratio of kilograms of weight to squared-meters of height. Healthy BMI is classified within the range [18.5,25), while [25,30) is classified as overweight, and BMI > 30 is classified as obese.

2 See e.g., Howard and Potter, 2012; Goetzel, et al., 2010; Ricci and Chee, 2005; Tsai et al., 2008; Burton, et al., 1999; Tucker and Friedman, 1998; Schmier, Jones, and Halpern, 2006; Finkelstein et al, 2010; Gates et al., 2008; Pronk et al., 2004

3 For a thorough review of the wage-BMI instrumental variables literature, see Kortt and Leigh, 2010.

4 Considering U.S. employees, Bhattacharya and Bundorf (2009) find evidence that wages for obese employees decrease as employers shift compensation to cover their higher insurance premiums, but Baum and Ford (2004) find that obese Americans with employer-provided insurance earn more than obese workers without it. Atella et al. (2008) also fail to find evidence for the insurance hypothesis in Europe.

5 Bozoyan and Wolbring (2011) and Wada and Tekin (2010) find evidence suggesting that women face a higher wage penalty than males when the measure is fat mass rather than BMI.

6 Dackehag, Gerdtham, and Nordin (2015) find that in Sweden, the obesity penalty is limited to males.

7 All results presented in Section 5 are robust to running a linear model on log wages rather than a generalized linear model with log link.

8 The obesity status of individual *i* is included in the proportional measures. Therefore, the proportional measure is of the work group as a whole, not “everyone else” in the work group.

9 The data do not contain any “blue collar” support personnel (e.g. custodians, security).

10 Cawley et al. (2015) note that non-classical measurement error in self-reported height and weight can upward bias the estimated effect of obesity on wages. Ljungvall, Gerdtham, and Lindbald (2015) indicate that misreporting of BMI is correlated with education. Bostrom and Diderichsen (1997) find that BMI computed from self-reported height and weight is systematically lower among females and obese individuals.

11 Race enters the equation as a binary white/nonwhite variable. Roughly two-thirds of non-whites are Asian, 10% are African American, and the remainder self-identify as Pacific Islander, Native American, or “Other.”

12 Data for C-Reactive protein and cholesterol are missing for 16 percent and 19 percent of the sample, respectively. Less than 1 percent of observations were missing data on heart rate. Since these are not the variables of interest and are assumed to be MAR, missing values were imputed using linear regression.

13 See e.g., Howard and Potter (2012); Holden et al. (2011); Niranjana et al. (2011); Goetzel, et al. (2009); Goetzel et al. (2004); Pronk et al. (2004); Aldana and Pronk (2001).

14 We concede that proxies for human capital accumulation may instead serve as a proxy for discrimination in promotion, and that measures of health may be endogenous to wages. However, this is true of productivity controls throughout the literature and is not a unique limitation of this study.

15 Parametric studies typically model the relationship between BMI and wages by utilizing a linear measure of BMI, or by creating a set of categorical BMI indicator variables (e.g., overweight, obese). Nonparametric estimates of the wage-BMI relationship have found the relationship to be nonlinear in multiple datasets (Kline and Tobias, 2008; Kan and Lee, 2012). For both males and females, the Akaike Information Criterion (Akaike, 1974) is lower using categorical indicators than a linear term indicating the indicators fit the data better.

16 Since the categories are in increments of $10,000, imputation could introduce potentially large measurement error. Therefore, this was not the primary specification of choice.
